# From Alpha-Thalassemia Trait to *NPRL3*-Related Epilepsy: A Genomic Diagnostic Odyssey

**DOI:** 10.3390/genes15070836

**Published:** 2024-06-25

**Authors:** Maryam Nabavi Nouri, Lama Alandijani, Kalene van Engelen, Soumitra Tole, Emilie Lalonde, Tugce B. Balci

**Affiliations:** 1 Division of Neurology, Department of Paediatrics, Schulich School of Medicine and Dentistry, Western University, London, ON N6A 5A5, Canadalama.alandijani@lhsc.on.ca (L.A.); 2 Children’s Health Research Institute, London Health Sciences Centre, London, ON N6A 5W9, Canada; 3 Division of Genetics, Department of Paediatrics, Schulich School of Medicine and Dentistry, Western University, London, ON N6A 5A5, Canada; 4 Division of Hematology and Oncology, Department of Paediatrics, Schulich School of Medicine and Dentistry, Western University, London, ON N6A 5A5, Canada; 5 Department of Pathology and Laboratory Medicine, Schulich School of Medicine and Dentistry, Western University, London, ON N6A 5A5, Canada

**Keywords:** refractory epilepsy, NPRL3, focal cortical dysplasia, hemoglobinopathy

## Abstract

**Introduction**: The *NPRL3* gene is a critical component of the GATOR1 complex, which negatively regulates the mTORC1 pathway, essential for neurogenesis and brain development. Located on chromosome 16p13.3, *NPRL3* is situated near the α-globin gene cluster. Haploinsufficiency of *NPRL3*, either by deletion or a pathogenic variant, is associated with a variable phenotype of focal epilepsy, with or without malformations of cortical development, with known decreased penetrance. **Case Description**: This work details the diagnostic odyssey of a neurotypical 10-year-old boy who presented at age 2 with unusual nocturnal episodes and a history of microcytic anemia, as well as a review of the existing literature on *NPRL3*-related epilepsy, with an emphasis on individuals with deletions who also present with α-thalassemia trait. The proband’s episodes were mistaken for gastroesophageal reflux disease for several years. He had molecular testing for his α-thalassemia trait and was noted to carry a deletion encompassing the regulatory region of the α-thalassemia gene cluster. Following the onset of overt focal motor seizures, genetic testing revealed a heterozygous loss of *NPRL3,* within a 106 kb microdeletion on chromosome 16p13.3, inherited from his mother. This deletion encompassed the entire *NPRL3* gene, which overlaps the regulatory region of the α-globin gene cluster, giving him the dual diagnosis of *NPRL3*-related epilepsy and α-thalassemia trait. Brain imaging postprocessing showed left hippocampal sclerosis and mid-posterior para-hippocampal focal cortical dysplasia, leading to the consideration of epilepsy surgery. **Conclusions**: This case underscores the necessity of early and comprehensive genetic assessments in children with epilepsy accompanied by systemic features, even in the absence of a family history of epilepsy or a developmental delay. Recognizing phenotypic overlaps is crucial to avoid diagnostic delays. Our findings also highlight the impact of disruptions in regulatory regions in genetic disorders: any individual with full gene deletion of *NPRL3* would have, at a minimum, α-thalassemia trait, due to the presence of the major regulatory element of α-globin genes overlapping the gene’s introns.

## 1. Introduction

The spectrum of genetic epilepsies has expanded beyond the well described ion channel dysfunction, with increasing discoveries of epilepsy genes with various functions, including chromatin remodeling, transcriptional regulation, and the regulation of the mechanistic target of the rapamycin (mTOR) protein. The GATOR1 complex, comprising DEPDC5 (DEP domain-containing protein 5), NPRL2 (nitrogen permease regulator-like 2), and NPRL3 (nitrogen permease regulator-like 3), plays a crucial role in inhibiting the mTOR signaling pathway, which is well known for its effects on cell growth and is essential for neurogenesis, synaptic transmission, and plasticity [1]. Haploinsufficiency of the genes (*DEPDC5*, *NPRL2*, *NPRL3*) that make up the GATOR1 complex has recently emerged as a prevalent cause of mostly focal epilepsies [2]. The *NPRL3* gene encodes a highly conserved and widely expressed protein located on chromosome 16p13.3 and overlaps the regulatory region of the α-globin gene cluster. Pathogenic variants of *NPRL3* can result in a loss of function within the GATOR1 complex, leading to excessive activation of the mTOR pathway [3]. Deletions on chromosome 16 involving the *NPRL3* gene have also been described as disease causing [4]. In this paper, we detail the presentation of a young boy with an atypical clinical picture; with focal seizures misdiagnosed as gastrointestinal symptoms and a genomic finding explaining both his epilepsy and systemic features, expanding our understanding of the genotypic landscape associated with *NPRL3* pathogenic variants.

## 2. Case Presentation

**Figure 1:**
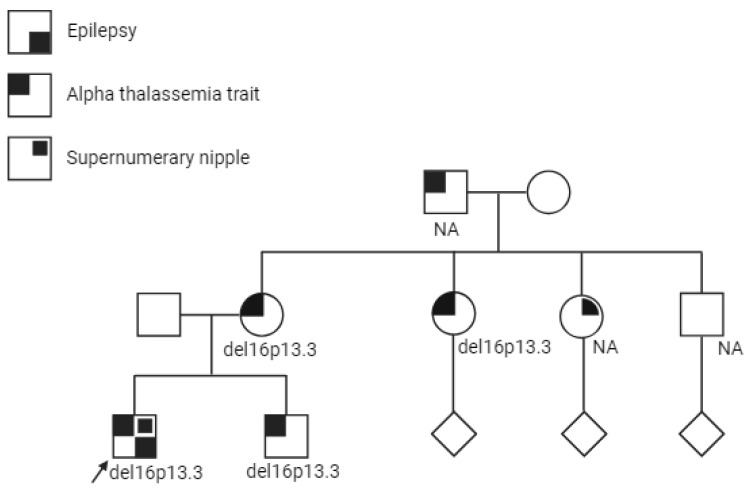
Pedigree of the family, with the proband shown with an arrow. Phenotypes depicted in the key in the top left corner. Cascade testing of the family revealed the deletion in multiple family members. NA: not available to test.

The male proband was born at 38 weeks of gestation to unrelated healthy parents of mixed European descent. Family history was significant for α-thalassemia trait in multiple individuals on the maternal side (Figure 1), however no one in the family had a confirmed molecular diagnosis. There was also no reported family history of epilepsy. Pregnancy and delivery were uneventful. He had neonatal jaundice requiring 24 h of phototherapy. He was diagnosed with gastroesophageal reflux as an infant, with frequent spit ups and vomiting in the first 6 months of life. He had neurotypical development. His past medical history also included two episodes of fever and myositis with high creatine kinase levels (resolved), recurrent nosebleeds, anaphylactic reactions (pistachio and cashew), and asymptomatic microcytic anemia (diagnosed as the familial α-thalassemia trait). Starting at age 2, he started having events involving watery eyes and repetitive swallowing. He was assessed by multiple medical services over the years, including gastroenterology, rheumatology, and hematology for different concerns, namely reflux, recurrent fevers, joint pain, and microcytic anemia. Molecular testing for α-thalassemia was sent to the provincial hemoglobinopathy laboratory, where testing for deletions and duplications in the α-globin gene cluster was performed using the multiplex ligation-dependent probe amplification (MLPA) method. The test showed a heterozygous large deletion, which causes carriers to exhibit microcytosis and hypochromia, with or without significant anemia (α-thalassemia trait). He was assessed by gastroenterology specialists for gastroesophageal reflux disease for several years, before he had his first overt focal motor seizure at age 7. Upon further clarification, the events that were treated as reflux, started with a “sweet taste” in his throat, followed by intense fear and a panicked facial expression, choking and mouthing movements, with repetitive truncal movements, followed by immediate recovery postictally, with no post-ictal aphasia. During the episodes, his awareness was maintained, but he was unable to articulate. At this stage, the episodes were highly suggestive of seizures originating from temporal/insular regions. They lasted less than a minute and occurred daily, mostly at night. Autoimmune workup was carried out and was unremarkable.

Physical examination at age 8 revealed four small hypopigmented macules, gray sclerae, bifid uvula, supernumerary nipple, and joint hypermobility. His neurological examination was unremarkable. There were no seizure risk factors. His echocardiogram was unremarkable. His interictal electroencephalogram (EEG) showed rare left mid-temporal discharges intermixed with slowing. His ictal EEG showed attenuation localizing mostly to left temporo-occipital localization, followed by the spreading of paroxysmal activity to the whole left hemisphere. His brain magnetic resonance imaging (MRI) was reported as normal. Carbamazepine (20 mg/kg/day) was not effective. Additional anti-seizure medications (ASM), including levetiracetam and eslicarbazepine, also failed to fully control his seizures.

**Figure 2:**
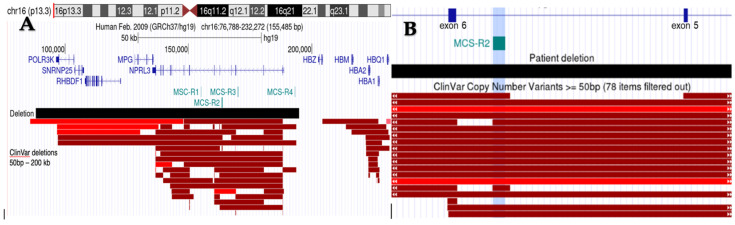
(**A**). Schematic of proband’s deletion involving the NPRL3 gene and the α-globin regulatory region on ss16p13.3. RefSeq are shown in blue, while the multi-species conserved (MSC) sites are shown in teal. The black box depicts the proband’s 106 kb deletion. Deletions submitted to ClinVar and ranging in size from 50 bp to 200 kp are shown in red, with dark red boxes corresponding to pathogenic or likely pathogenic deletions, and bright red boxes for variances of unknown significance (VUS) as determined by the original submitters. Deletions overlapping MCS-R2 result in significantly reduced α-globin gene expression and are considered pathogenic for α-thalassemia, regardless of the overlap with the α-globin gene cluster. (**B**) Zoomed in region of *NPRL3* intron 5 (NM_001077250.3), with the MCS-R2 region highlighted in blue. White arrows within the ClinVar deletions indicate that the deletions continue beyond the region captured.

A heterozygous full gene deletion of the *NPRL3* gene was detected on a multigene next-generation sequencing epilepsy panel, establishing the diagnosis of *NPRL3*-related epilepsy (NRE). Follow-up studies with a chromosomal microarray revealed a maternally inherited, pathogenic 106 kb deletion on 16p13.3 (88165-194845) (hg19), resulting in the whole gene loss of *NPRL3* and *POLR3K* (Figure 2). This particular region also overlaps with a regulatory element of the α-globin gene cluster, MCS-R2, but does not include the actual α-globin genes. Given the association of focal cortical dysplasia with NRE, postprocessing of the MRI images revealed left hippocampal sclerosis and left mid-posterior para-hippocampal focal cortical dysplasia (FCD) [5]. His functional MRI suggested left hemispheric dominance for language function. The patient was placed in the epilepsy surgery pathway and considered for mTOR inhibitors.

## 3. Discussion

**Figure 3:**
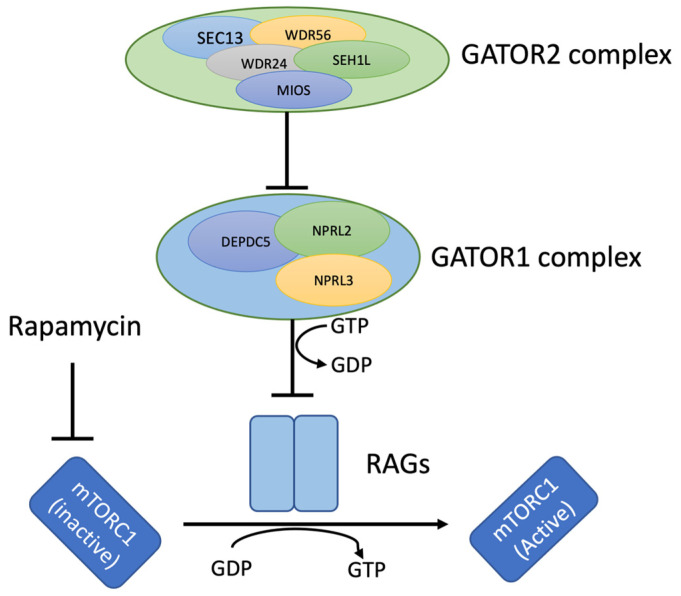
Intracellular signaling pathways demonstrating the mTORC1 and GATOR complex regulation. The GATOR1 protein complex consists of NPRL3 (nitrogen permease regulator-like 3), DEPDC5 (DEP domain-containing protein 5), and NPRL2 (nitrogen permease regulator-like 2) and exerts an inhibitory effect on the mTORC1 pathway. Rapamycin is a well-known drug to inhibit.

The *NPRL3* gene encodes a component of the GATOR1 complex, which plays a crucial role in inhibiting the mTORC1 pathway, a key regulator of cellular processes vital for cell growth, neurogenesis, and brain development (Figure 3) [1,6]. Pathogenic variants in *NPRL3*, mostly loss-of-function or truncating mutations, like nonsense and frameshift variants, cause a spectrum of epilepsy phenotypes collectively termed *NPRL3*-related epilepsy (NRE) [1,7,8]. The severity of epilepsy phenotypes is associated with changes in the protein function or levels, and NRE is well-known to have decreased penetrance in families [3]. Although multiple individuals in this family had a clinical history of α-thalassemia trait, there was no reported history of epilepsy. The proband’s mother and brother were both confirmed to carry the familial deletion and are seizure free. This is consistent with the reduced penetrance seen in NRE. In the largest pedigree thus far reported of NRE (*n* = 133) among an Old Order Mennonite kindred [9], 36.1% of subjects had a history of seizures, with an estimated penetrance of 28%. Affected individuals displayed markedly heterogeneous epilepsy phenotypes, despite sharing the common truncating founder *NPRL3* variant (c.349delG, p.Glu117Lysfs) [9].

The seizure phenotypes in patients with NRE are diverse and vary across different studies. In one study, among 74 patients with NRE [10], there was a predominance of affected females (63.8%). The mean age at seizure onset was 8.5 years (range: 1 day to 51 years of age). Developmental delays, language delays, and mild cognitive impairment affected only a small number of cases. The most common seizure type was focal-onset seizures, as observed in 90% of patients. Of these, frontal lobe onset was the predominant seizure onset pattern (37%), followed by unspecified focal onset (28%), temporal lobe onset (13%), frontotemporal onset (10%), and central onset (9%) [10]. Secondary generalized seizures occurred in 13.5% of patients, while 12% had primary generalized seizures without a clear focal onset. Infantile spasms have also been reported as part of the phenotypic spectrum of NRE [9,11]. Epilepsy syndromes that have been described in patients with NRE include sleep-related hyperkinetic epilepsy (SHE) and familial focal epilepsy with variable foci (FFEVF) [6,10].

*NPRL3* is located on chromosome 16p13.3, which is adjacent to the α-globin gene cluster, responsible for α-thalassemia when mutated or deleted [6]. In addition, the regulatory region controlling the expression of α-globin genes is located within the intronic region of *NPRL3.* There are four multispecies conserved regulatory sequence (MCS-R) elements upstream of the α-globin gene, with MCS-R2 known to be the major regulatory element for α-globin gene expression [12,13,14]. MCS-R2 is also known as hypersensitivity site 40 or HS-40, and located within intron 5 of *NPRL3* (MANE transcript, NM_001077250.3) Therefore, large *NPRL3* deletions or structural variants in this region of chromosome 16 can potentially disrupt both the *NPRL3* gene, leading to epilepsy, as well as the α-globin genes, resulting in the α-thalassemia trait, or more severe forms if a second α-thalassemia variant is present on the other chromosome [7,11].

To our knowledge, there has only been one other publication [11] that has demonstrated the co-occurrence of NRE and the α-thalassemia trait due to deletion. In this report, the deletion was larger than our case, 552 kb at 16p13.3 (93722-646006, hg19) and included whole gene deletion of both *NPRL3* and *HBA* (hemoglobin subunit α) 1 and 2, as opposed to just the regulatory region in our case. This patient presented with infantile spasms and had a left hemispheric cortical malformation. Globin *HBA1*/*HBA2* testing in this individual confirmed the cis deletion (αα/--) consistent with the α-thalassemia trait. Our patient’s globin gene testing, as expected, did not detect the whole *HBA* gene deletion, but did identify the regulatory region MCS-R2 deletion, which has previously been shown to result in reduced α-globin expression and is associated with autosomal recessive α-globin disorders [15] (Figure 3). Our case highlights that the overlap of the α-globin regulatory region MCS-R2 with the *NPRL3* gene may be overlooked when performing hemoglobinopathy testing, due to the gene-centric nature of the assay. However, given that MCS-R2 is located within intron 5 of *NPRL3*, any individual with an α-globin regulatory region deletion should be considered for further testing (for example, with a chromosomal microarray) to define the breakpoints of the deletion and to assess their *NPRL3* status. Conversely, any individual with full gene deletion of *NPRL3* would consequently be (at least) a carrier of α-thalassemia, due to the co-occurring deletion of MSC-R2. Clinical laboratories performing hemoglobinopathy testing or chromosomal microarrays should consider including the information about this overlap in their reporting if such deletions are detected, for the ordering clinicians to consider either diagnosis, keeping in mind that *NPRL3*-related epilepsy has decreased penetrance. This phenomenon has major counselling implications, both for the individual’s own health and for family planning purposes.

This patient’s epilepsy phenotype did not fit into epilepsy syndromes that were described in the literature. Despite the majority of his seizures being reported during sleep, there were rare daytime seizures. Beginning at an early age, the seizures manifested as nighttime episodes of a sweet taste in his throat, followed by intense fear, choking, repetitive mouthing, and truncal movements, while he remained aware, but unable to articulate. Initially, these symptoms were misattributed to reflux, resulting in a delay in diagnosis, until he experienced his first bilateral tonic–clonic seizure. Furthermore, the familial microdeletion on chromosome 16p13.3, with its impact on neighboring genes, was not recognized until a concrete epilepsy diagnosis was made in our proband, further delaying the accurate counselling of the family. Most importantly, despite an initially unremarkable clinical MRI, the images underwent advanced postprocessing analysis at the Montreal Neurological Institute (MNI), using texture pipeline analysis. This decision was influenced by the patient’s diagnosis of *NPRL3* deletion, known to be associated with malformations of cortical development. The postprocessing analysis revealed focal cortical dysplasia in the left parahippocampal region, as well as subtle left hippocampal sclerosis. These findings aligned well with the scalp EEG findings, indicating a deep generator with delayed spread to the frontal insular lobe, explaining the seizure semiology.

The spectrum of brain malformations associated with mTOR pathway dysregulation ranges from small FCDs to hemimegalencephaly (HME) [16]. A recent study reviewed a total of 116 cases of NRE, wherein brain MRI results were available from 68 cases, revealing 26 cases with an abnormal MRI [6]. Malformation of cortical development (MCD) was diagnosed in 23 patients, focal cortical dysplasia (FCD) in 18, hemimegalencephaly in 3, and periventricular heterotopia and polymicrogyria (PMG) in 1 patient each. From the 18 cases who presented with FCD, together with our case, we summarize their presentation, latency to diagnosis and treatment response, and lesion distribution in Table 1. FCD IIa (eight cases) and FCD IIb (one case) were confirmed by histology examinations in a subset of 10 patients who underwent epilepsy surgery [6]. A systematic literature review revealed that 60% of patients with GATOR1 complex gene variants, including *NPRL3*, achieved seizure freedom following epilepsy surgery. Focal cortical dysplasia (FCD) type IIa was similarly identified as the pathology in these cases [17]. From the cohort summarized in Table 1, 9/10 patients whose follow-up surgical data was available to us had an Engel class I/II seizure outcome. Additionally, *NPRL3* pathogenic variants are among the genetic causes of bottom-of-sulcus dysplasia [16,18]. The success of epilepsy surgery often depends on the precise localization of the epileptogenic zone and the presence of FCD, which is frequently associated with *NPRL3* mutations. The presence of FCD can influence both the decision to perform surgery and the surgical outcomes [17]. Recently, laser interstitial thermal therapy has been highlighted as an effective treatment for drug-resistant NRE, offering better tolerance and less morbidity compared to traditional open surgical resection. This method is particularly beneficial in cases involving multiple seizure foci, leading to the cessation of seizures [19].

Precision treatment with mTOR inhibitors, such as sirolimus, holds promise in treating NRE due to its ability to counteract the abnormal activation of the mTOR signaling pathway caused by *NPRL3* gene variations. However, in two previously reported cases [11,23], sirolimus did not achieve sustained seizure control. One patient initially responded well during the first three months of treatment but had to discontinue sirolimus due to side effects like intermittent diaper rashes, eczema, and respiratory infections. Sirolimus, however, could still be considered as a bridging therapy until surgery can be performed, especially in patients who are non-responsive or only partially responsive to other antiepileptic drugs. mTOR inhibitors have not yet been trialed in our patient, mainly due to the side-effect profile and scarcity of evidence surrounding their effectiveness.

## 4. Conclusions

While the primary focus of *NPRL3* research has been on its role in epilepsy, it is recognized that if this gene and surrounding regions are deleted, α-thalassemia phenotypes can co-occur, given its genomic location near the α-globin gene cluster on chromosome 16p13.3. We add to the body of literature that individuals with *NPRL3* whole gene deletion are consequently α-thalassemia carriers, due to the genomic overlap of a major α-globin regulatory region and *NPRL3*’s intron 5. Similarly, an individual with an α-globin regulatory region deletion may be at risk of developing epilepsy, due to *NPRL3* disruption. Non-coding DNA mutations, such as regulatory region disruptions, typically escape traditional sequencing methods, as the genes they regulate remain intact. They are a more recent and increasingly recognized cause of “unsolved” human genetic disease, making their detection impactful [24]. Our patient’s diagnostic odyssey is a prudent example of how recognizing patterns and using a multidisciplinary approach, using all the available molecular techniques in the neurologist’s toolbox, enables an accurate and complete diagnosis of a molecularly complex presentation, further complicated by the decreased penetrance and clinical variability of this type of genetic epilepsy. Another important aspect of this case is the identification of FCD, which was initially overlooked, and is increasingly recognized as able to change outcomes of patients with drug-resistant epilepsy, which highlights the importance of more sophisticated imaging tools, as well as the meticulousness of the search for lesions in patients with NRE. Due to the genetic and phenotypic heterogeneity of NRE, treatments are often tailored based on clinical presentations observed in patients. This approach helps in formulating the most optimal management plan, based on an accurate understanding of the underlying genetic and structural abnormalities

## Figures and Tables

**Table 1:** Genetic and clinical features of subjects with *NPRL3*-related epilepsy and focal cortical dysplasia. NPRL3 DNA variants are reported in relation to the NM_001077350.3 reference sequence.

No.	NPRL3 cDNA Variant and Protein Alteration	Age of Seizure Onset	Sex	Seizure Type	Time to Clinical Diagnosis	Time to Genetic Diagnosis	No. of ASM	Histopathology	Radiological Distribution of FCD	Type of Surgery	Epilepsy Surgery Outcome	Comorbidities
1	NPRL3:c.(?_-21)_(*21_?)del(arr[hg19] 16p13.3(88165_194845)x1) Full gene deletion	2 years	M	Left temporal plus epilepsy	5 years	2 years	3	NYD	Left mid-posterior parahippocampal dysplasia	Pending	N/A	Joint hypermobility
2 [20]	c.1375_1376dupAC, p.(S460Pfs*20) Frameshift mutation	0 months	M	Left temporal epilepsy and bilateral clonic seizures	A few days	N/A	N/A	FCD IIa	Right posterior quadrantic dysplasia	Temporo-parieto-occipital disconnection and frontocentral corticectomy at 11 weeks	Engel II: Partial seizure control at 3 years on oxcarbazepine (residual dysplasia)	Global developmental delay, left hemiplegia, and hemianopia
3 [20]	c.1375_1376dupAC p.(S460Pfs*20) Frameshift mutation	7 years	F	Right focal lobe epilepsy	N/A	N/A	2	FCD IIa	Bottom-of-sulcus dysplasia in the right anterior cingulate sulcus	Resection of FCD at 7 years of age	Engel I: Seizure free at one year after surgery on oxcarbazepine	None
4 [20]	c.1352-4delACAG insTGACCCATCC p.(?) Splicing mutation	4 months	M	Bilateral tonic and orofacial motor manifestations	N/A	N/A	3	FCD IIa	Extensive left frontal operculum and insula dysplasia	Staged resections at 6 and 7 months. Residual dysplasia from the left insula and frontal operculum was resected at 4 years	Engel I: Seizure free and off medication at 6 years	Near normal cognitive and language development and right hemiparesis
5 [20]	c.275G > A p.(R92Q) Missense mutation	15 months	F	Right frontal lobe epilepsy	N/A	N/A	2	FCD IIa	Diffuse dysplasia in the left central head region	Resection was performed at 23 months	Engel I: At 3 years, seizure free and weaning ASM	Mild right hemiparesis and language delay
6 [2]	c.1270C > T p.(R424*) Nonsense mutation	2 months	NA	Left frontal lobe epilepsy (FFEVF)	2 months	N/A	4	FCD IIa and hippocampal sclerosis	Left frontal lobe FCD	2 years: postoperative MRI with incomplete resection of FCD, left hippocampal atrophy	Engel II: incomplete FCD resection at 1 year and second surgery at 5 years: rare seizures when medication errors occurred	N/A
7 [2]	c.1070delC p.(P357Hfs*56) Frameshift mutation	2 years	NA	Right frontal lobe epilepsy (FFEVF)	N/A	N/A	2	FCD IIb	Right frontoparietal FCD	No	Engel I: Seizure free at 6 months	N/A
8 [21]	c.973_975del p.(I325del) Inframe deletion	0 months	F	Left frontal lobe epilepsy	N/A	N/A	2	FCD IIa	Left frontal lobe FCD	Resective surgery	Engel I: Seizure free at 1 year 3 months	Moderate ID and impaired motor development
9 [22]	c.48delG p.(S17Afs*70) Frameshift mutation	2 years	M	Right Frontal lobe epilepsy	3 years	Post-resection	1	FCD IIa	Right postero-mesial frontal FCD	Resection at 6 years of age	Engel III: focal seizures returned at 10 years of age (residual dysplastic)	Inattention and distractibility
10 [22]	c.48delG p.(S17Afs*70) Frameshift mutation	6 weeks	M	Left frontal lobe epilepsy	6 weeks	Post-resection	1	FCD IIa	Left anteromesial frontal FCD	Focal resection at 2 years	Engel I: seizure free postoperatively, and medication was withdrawn at 3 years	None
11–18 [9]	c.349delG p.(E117Kfs*5) Frameshift mutation	0 months–15 years	N/A	All had focal onset epilepsy	No single-patient data available							
19 [6]	c.1174C > T p.(Q392*) Nonsense mutation	10 days of age	F	Epileptic spasms and focal seizures	A few days	1 year	7	N/AN/AN/A	Abnormal signal over left lateral ventricle and a widened left frontotemporal sulcus	Hemispherectomy at 1 year and 2 months of age	Engle I at year	Right-sided cerebral palsy

ASM: anti-seizure medication, FCD: focal cortical dysplasia, FFEVF: familial focal epilepsy with variable foci.

## Data Availability

The original contributions presented in the study are included in the article, further inquiries can be directed to the corresponding author.
